# The Pea *R2R3-MYB* Gene Family and Its Role in Anthocyanin Biosynthesis in Flowers

**DOI:** 10.3389/fgene.2022.936051

**Published:** 2022-07-06

**Authors:** Yating Yang, Zhuo Yuan, Conghui Ning, Baoling Zhao, Ruoruo Wang, Xiaoling Zheng, Yu Liu, Jianghua Chen, Liangliang He

**Affiliations:** ^1^ School of Life Sciences, Division of Life Sciences and Medicine, University of Science and Technology of China, Hefei, China; ^2^ CAS Key Laboratory of Topical Plant Resources and Sustainable Use, CAS Center for Excellence in Molecular Plant Sciences, Xishuangbanna Tropical Botanical Garden, Chinese Academy of Sciences, Kunming, China; ^3^ University of Chinese Academy of Sciences, Beijing, China; ^4^ College of Life Science, Southwest Forestry University, Kunming, China

**Keywords:** *Pisum sativum* L., *R2R3-MYB* gene family, flower color, anthocyanin biosynthesis, tissue-differential expression

## Abstract

Pea (*Pisum sativum* L.) is one of the most important legume crops in the world, and it has attracted great attention for its high nutritive values. Recently, the crop breeding program has been focused on the crop metabolic engineering (i.e., color, flavor, nutrition) to improve the quality of crop. As a major group of transcription factors forming the ternary MYB–bHLH–WD repeat protein (MBW) complex to regulate the anthocyanin biosynthesis pathway, members of *R2R3-MYB* gene family have always been the focus of research targets to improve the valuable metabolic product of crops. Until now, few report about the *R2R3-MYB* gene family of pea has been released. In this study, we identified 119 *R2R3-MYB* genes in the assembled pea genome (Version 1a), of which 111 were distributed across 14 chromosomes. Combining with the 126 R2R3-MYB protein sequences of *Arabidopsis*, we categorized 245 R2R3-MYB proteins into 36 subgroups according to sequence similarity and phylogenetic relationships. There was no member from subgroup 12, 15 and 29 existing in pea genome, whereas three novel subgroups were found in pea and named as N1-N3. Further analyses of conserved domains and Motifs, gene structures, and chromosomal locations showed that the typical R2 and R3 domains were present across all R2R3-MYB proteins, and Motif 1, 2, and 3 were identified in most members. Most of them had no more than two introns. Additionally, 119 pea *R2R3-MYB* genes did not experience large-scale duplication events. Finally, we concluded that several candidate genes may be responsible for the spatiotemporal accumulation of anthocyanins in pea petals. *PsMYB116* was predominantly expressed in the dorsal petals to presumably activate the anthocyanin biosynthesis pathway, while *PsMYB37* and *PsMYB32* may positively regulates the anthocyanin accumulation in the lateral petals. This study not only provides a good reference to further characterize the diverse functions of R2R3-MYB genes but also helps researchers to understand the color formation of pea flowers.

## Introduction

Pea (*Pisum sativum* L.) is the second most important grain legume in the world after soybean and it plays a significant role in the discover of laws of genetics since the 18th century (Kreplak et al., 2019). Pea possesses many important agronomic traits and is rich in dietary protein, complex starch, fiber and flavonoids which are beneficial to human health (Guillon and Champ, 2002). Anthocyanin is one of the widely distributed pigments in flowers which can help plants to attract pollinators, and protect plants against varies biotic and abiotic stresses (Gould, 2004; Buer et al., 2010; Nishihara and Nakatsuka, 2011; Zhao and Tao, 2015).

Most cultivars of peas have white flowers, while the wild peas have purple flowers which is determined by anthocyanin (Hellens et al., 2010). The regulation mechanism of anthocyanin biosynthesis is highly conserved and has been well studied in many plants. As key regulators, R2R3-MYBs play critical roles in regulation of anthocyanin biosynthesis *via* consisting the MBW complexes to control the expression of target genes (Espley et al., 2007; Gonzalez et al., 2008). Among MBW complex, R2R3-MYB proteins usually serve as transcriptional activators *via* specific recognition of target genes; however, there are many evidences to show that some R2R3-MYBs function as transcriptional repressors by interacting with bHLH protein to compete with R2R3-MYB activators or by recruiting other repressors to inhibit the transcription activation of MBW complex (Zhou et al., 2018; Li et al., 2020b; Zhong et al., 2020; Wang et al., 2021).

Many R2R3-MYBs have been extensively studied and shown to regulate tissue-specific anthocyanin accumulation in plants. For instance, *AtPAP1* can enhance the expression of anthocyanin biosynthetic genes in *Arabidopsis* (Borevitz et al., 2000). The *Aft* (*anthocyanin fruit*) combined with the recessive *atv* results in light-dependent accumulation of anthocyanins in fruit skin of “Indigo Rose” (Sun et al., 2020). *DcMYB113*, which is specifically expressed in roots, determines the accumulation of anthocyanins in purple carrots (Xu et al., 2020). *AhTc1* encodes an R2R3-MYB transcription factor and determines purple testa color in peanut (Zhao et al., 2019). GmMYBA2 interacts with GmTT8a to activate the expression of anthocyanin biosynthetic genes in soybean seed coats (Gao et al., 2021). For ornamental plants, a member of R2R3-MYBs was found to determine the formation of different pigmentation patterns in flowers. In petunia, *An2* controls anthocyanin accumulation in the corolla and tube (Francesca Quattrocchio et al., 1999). *PcPAP1* and *PcPAP2* are responsible for the unbalanced pigment distribution in orchids (Li B.-J. et al., 2020a). *FhMYB27*, *FhMYBx*, and *FhPAP1* are reported to participate in flower coloration in *Freesia hybrids* (Li et al., 2020b; Li et al., 2020c). These studies suggest that the mechanisms of R2R3-MYBs regulating anthocyanin biosynthesis appear to be fairly sophisticated, but it is remarkable that some key R2R3-MYBs are conserved among many dicotyledons plants, and members of subgroup six of the R2R3-MYBs family are considered as the key factor to positively regulate the anthocyanin biosynthesis in different tissues.

In pea, numerous loci are reported to be responsible for the regulation of anthocyanin biosynthesis in different tissues. Six major loci including *A*, *B*, *Ar*, *Cr*, *Am*, and *Ce* had been shown to play important roles in the color formation of pea flowers. There are also a number of loci that determine the accumulation of pigmentation in other tissues in pea, including *Gp*, *Pu* and *Pur* which are associated with the formation of pod pigments (Statham et al., 1972b). The *A* locus was necessary for the expression of *CHS1* in petals, and neither a/a mutant nor a2/a2 mutant had the phenotype of anthocyanin accumulation in petals (Harker and Coen, 1990). Molecular cloning of the *A* locus revealed that it encodes a bHLH transcription factor functioning to determine white flowers (Hellens et al., 2010). *MYB26* was the first reported *MYB*-like gene which strongly expressed in flower and may regulate phenylpropanoid production (Uimari and Strommer, 1997). Although these studies have initially explored the formation of pigments, roles of R2R3-MYBs in anthocyanin biosynthesis regulation have not been systematically studied in pea flowers.

MYB transcription factors represent one of the largest plant transcription factor families (Jiang and Rao, 2020). The MYB family harbors a characteristic conserved DNA-binding domain named as imperfect amino acid sequence repeats (R) at the N-terminus and a relatively diverse C-terminus. Each repeat typically contains approximate 52 amino acid residues, among which three regularly spaced tryptophan residues form three a-helices (Kanei-Ishii et al., 1990). The second and third helices of each repeat constitute a helix–turn–helix (HTH) structure which forms a hydrophobic core in three-dimensional structure (Ogata et al., 1996). The third helix directly intercalates in major groove and contacts with the target DNA sequences, which named as “recognition helix” (Ogata et al., 1992). In contrast, MYB proteins do not feature highly conserved sequences in their C-terminal, providing multi-functionality of plant MYB proteins in different subgroups (Kranz et al., 1998). As a result, the second and third helix determine the sequence-specific DNA binding properties of MYB transcription factors, while the low-conserved Motifs in C-terminal regions determine the diversity function of R2R3-MYBs. Alternatively, the second and third helixes may share relatively more conservation than the first helix structure in each repeat (Jin and Martin, 1999).

Based on the number of adjacent repeats, MYB proteins are divided into different classes, namely, 1R-MYBs (R1/R2/R3-MYBs and MYB-related proteins), R2R3-MYBs (2R-MYBs), R1R2R3-MYBs (3R-MYBs) and 4R-MYBs (R1R2R2R1/2-MYBs). R2R3-MYBs constitute the largest family of MYB transcription factors (Ogata et al., 1996; Dubos et al., 2010). The number of *R2R3-MYB* genes apparently increased during plant evolution. For example, 22, 41 and 50 *R2R3-MYB* genes have been identified in *Klebsormidium nitens*, *Selaginella moellemdorffii*, and *Physcomitrella patens*, respectively (Jiang and Rao, 2020; Pu et al., 2020), and 109, 126, 244, 256 and 393 *R2R3-MYB* genes have been found in *Oryza sativa*, *Arabidopsis thaliana*, *Glycine max*, *Brassica rapa* ssp. *pekinensis*, and *Fragaria × ananassa*, repectively (Yanhui et al., 2006; Dubos et al., 2010; Du et al., 2012b; Wang et al., 2015; Liu et al., 2021). However, little is known about the composition of the *R2R3-MYB* gene family in pea and their roles in the regulation of flower pigmentation pattern.

Now, the available chromosome-scale reference genome of pea made it possible to analyze the R2R3-MYB transcription factor family (Kreplak et al., 2019). Here, we performed a genome-wide analysis of the *R2R3-MYB* gene family and characterized several candidate genes that may play roles in flower anthocyanin accumulation. We first identified 119 *R2R3-MYBs* in pea genome and analyzed their subgroup constitutions, gene structures, conserved Motifs and chromosomal distributions. Next, the anthocyanin accumulation of purple flowers during different developmental stages in pea was investigated. The expression patterns of subgroup six *R2R3-MYBs* in different petals during flower development were also determined. As far as we know, this is the first report to analyze the *R2R3-MYB* gene family in pea, and this study not only provides a good reference to further characterize the functions of *R2R3-MYB* genes but also helps researchers to understand their diverse functions of *R2R3-MYBs*.

## Results

### Identification of Members of 119 *R2R3-MYBs* in Pea

To fully understand the *R2R3-MYB* gene family in pea, we searched the whole-genome sequence for genes containing the MYB domain by using HMM search, and then the sequences with incomplete R2R3-MYB domain structures were removed *via* Pfam database searching. Sequences were further checked by carrying out a multiple sequence alignment. Finally, 119 typical R2R3-MYB transcription factors were identified, and according to the positions of these transcription factors on the chromosomes, we named them PsMYB1 ∼ PsMYB119 (Supplementary Table S1).

To further characterize the R2R3-MYB proteins, the physiochemical properties, including protein length, molecular mass and isoelectric point value were analyzed (Supplementary Table S1). We found that the proteins varied from 166 to 677 amino acids in length with an average of 316.6 amino acids, and 76% of them were between 250 and 350 amino acids. The estimated molecular mass ranged from 19.28 to 75.72 kDa, and PsMYB117 had the longest length of 677 amino acids and the highest molecular mass of 75.72 kDa, while PsMYB97 had the shortest length of 166 amino acids and the lowest molecular mass of 19.28 kDa. The predicted isoelectric points of the proteins varied from 4.64 to 9.59, among which 64.7% of proteins had an acidic isoelectric point of less than 7, and the rest possessed a basic isoelectric point of more than 7.

### Phylogenetic Analysis and Classification of the *R2R3-MYB* Gene Family

**FIGURE 1:**
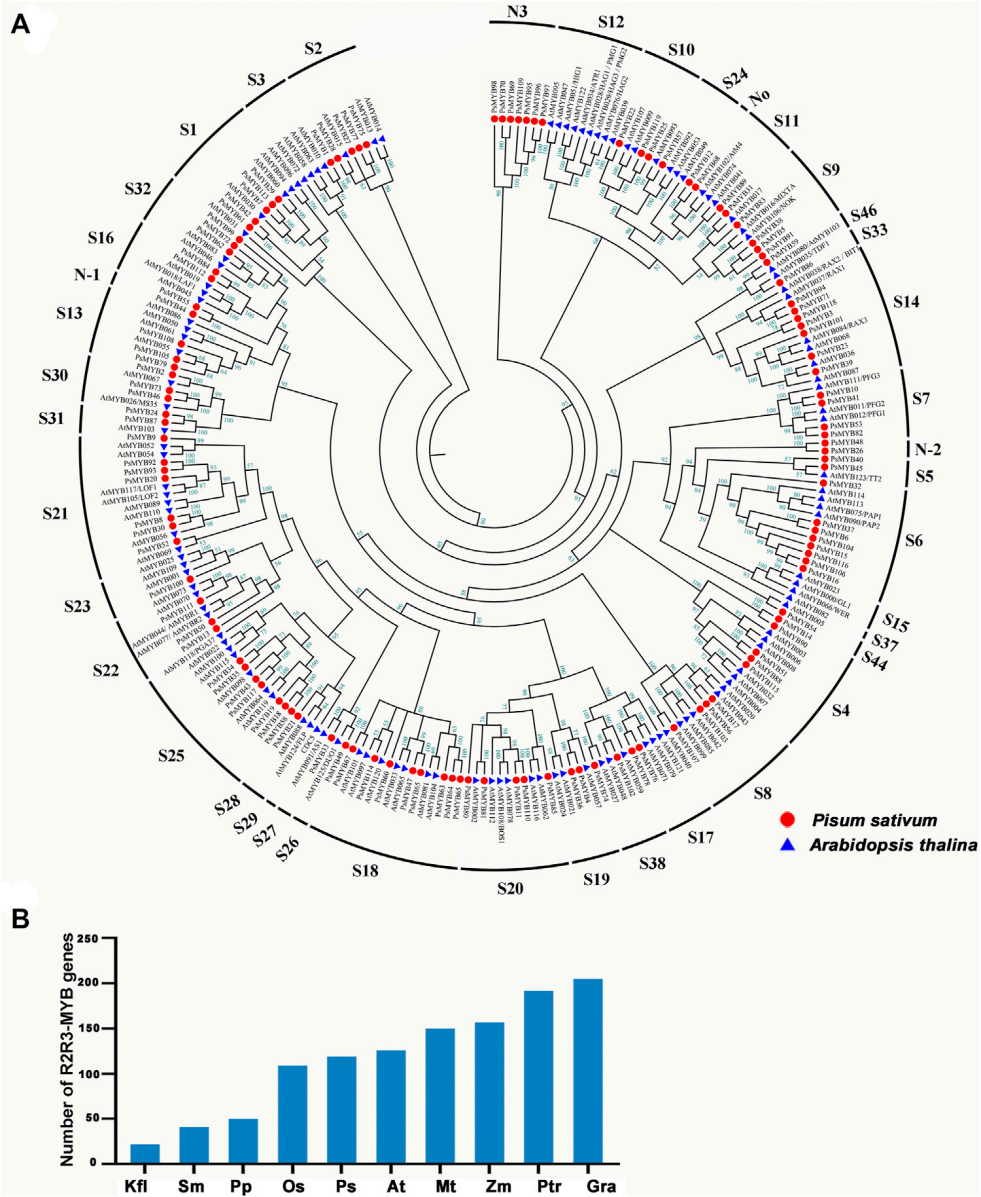
Phylogenetic and evolutionary analysis of the *R2R3-MYB* gene family in different plant species. **(A)** The phylogenetic tree of *R2R3-MYB* genes in *A*. *thaliana* (At), *P.sativum* (Ps). Thirty-six subgroups were obtained, namely, S1, S2, S3, S4, S5, S6, S7, S8, S9, S10, S11, S13, S14, S16, S17, S18, S19, S20, S21, S22, S23, S24, S25, S26, S27, S28, S30, S31, S32, S33, S38, S44, S46, N1, N2, N3. The genes that belonged to the same organism were marked in the same color. **(B)** Comparisons of *R2R3-MYB* genes number across a wide range of plant species. Kfl, *Klebsormidium nitens*; Sm, *Selaginella moellemdorffii*; Pp, *Physcomitrella patens*; Os, *Oryza sativa*; Ps, *Pisum sativum.* L; At, *Arabidopsis thaliana*; Mt, *Medicago truncatula*; Zm, Zea mays; Ptr, *Populus trichocarpa*; Gra, *Gossypium raimondii*.

To investigate the evolutionary relationships between *R2R3-MYB* genes, a total of 256 R2R3-MYB protein sequences consisting of 126 members from Arabidopsis and 119 members from pea were used to generate an unrooted phylogenetic tree. Referring the results of Dubos et al. (2010), Du et al. (2015) and Jiang and Rao (2020), the *R2R3-MYB* genes of the two species were divided into 41 subfamilies (Figure 1A; Supplementary Table S2). The pea *R2R3-MYB* genes were clustered into 36 subfamilies, and most subfamilies (i.e., S1–S11) contained members from both pea and *Arabidopsis*, but the number varied between pea and *Arabidopsis*. The S3, S23–S24, and S26–S28 subfamilies have only one member in pea but multiple members in *Arabidopsis*, while the S1 and S6 subfamily had more members in pea. These indicated that the expansion of some R2R3-MYB subfamilies has occurred independently in pea and *Arabidopsis*. We also identified several species-specific MYB subgroups, including three novel subfamilies (Novel-1, Novel-2, and Novel-3) are pea-specific, and some subfamilies (i.e., S12, S15, S29(CDC5) and S37) are *Arabidopsis*-specific. These results mean that these gene subfamilies must have occurred after the divergence of *Arabidopsis* and pea. The members clustered in the same subgroup usually exhibit a similar function. For example, S4, S5, S6, and S7 subgroups participate in phenylalanine metabolism, and S6 subgroup is thought to be involved in anthocyanin synthesis pathway. For instance, S4 subgroup members inhibited PA synthesis in *Arabidopsis*, including AtMYB7, AtMYB32, AtMYB4, AtMYB3, AtMYB6, and AtMYB8, indicating that the member in S4 in pea may have the same function in PA synthesis. PAP1(AtMYB75), PAP2(ATMYB90), AtMYB113, and AtMYB114 were known to be involved in anthocyanin biosynthesis, suggesting that these eight R2R3-MYBs may control anthocyanin biosynthesis in pea.

We searched for *R2R3-MYB* genes in 10 species ranging from lower aquatic plants to higher terrestrial plants to determine the origin of the *R2R3-MYB* gene (Yanhui et al., 2006; Dubos et al., 2010; Du et al., 2012a; He et al., 2016; Li et al., 2019; Jiang and Rao, 2020; Pu et al., 2020) According to this analysis, *R2R3-MYB* gene is present in all researched plant and there has been a tendency toward an increase in gene number over the course of plant evolution with a dramatically increase in angiosperms (Figure 1B), suggesting that the *R2R3-MYB* gene family is expanded during evolution to overcome the increased biological complexity.

### Conserved Sequence Analysis of R2R3-MYB Proteins

**FIGURE 2:**
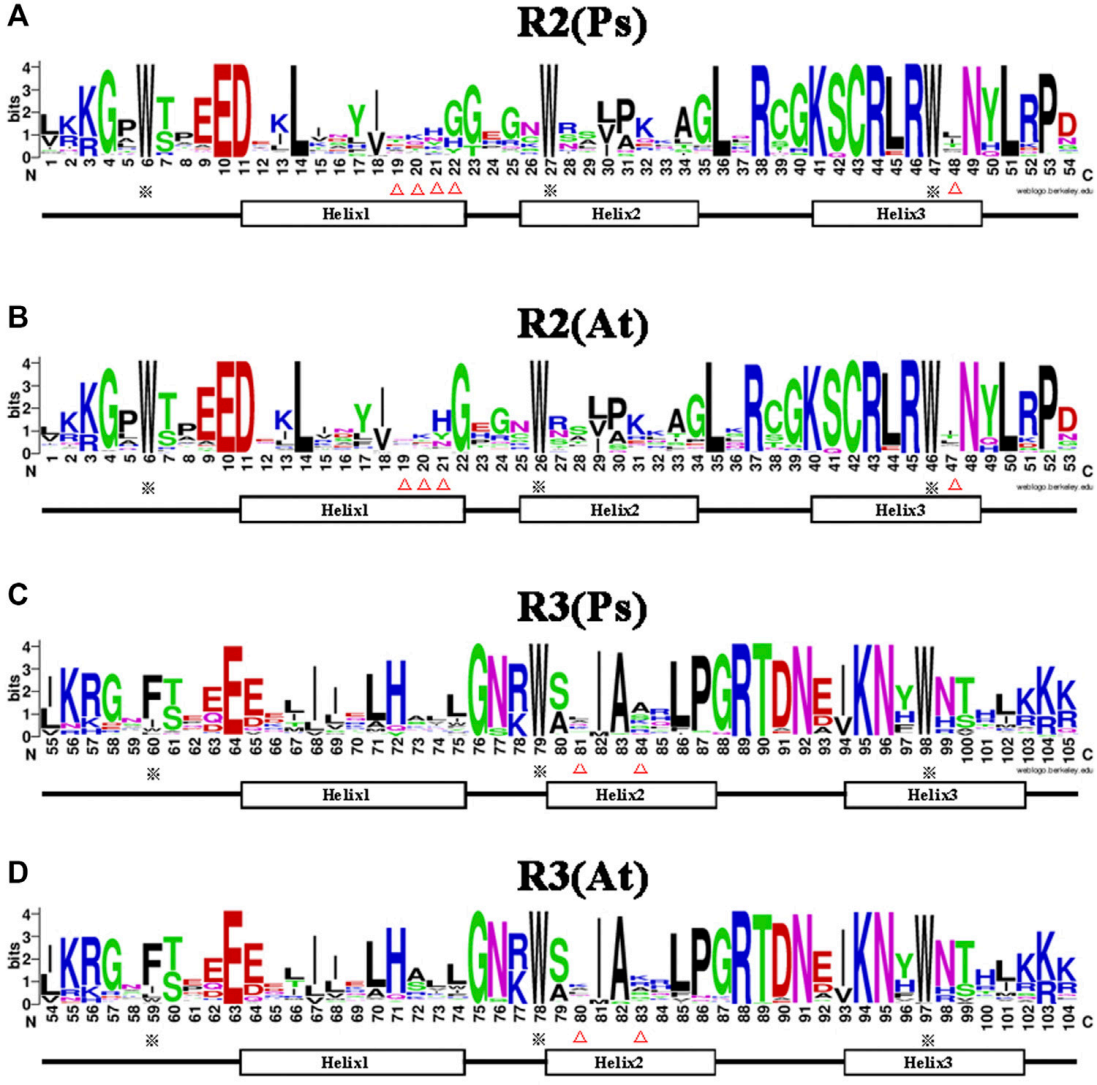
Comparison of the DNA-binding domains in R2R3-MYB proteins of pea and *Arabidopsis*. **(A,B)** Sequence logos showing the first part (R2) of the DNA-binding domains in pea **(A)** and *Arabidopsis* **(B)**. **(C,D)** The second part (R3) in pea **(C)** and *Arabidopsis* **(D)**. MYB repeats are based on full-length alignments of all R2R3-MYB domains in these two species. The bit score indicates the information content for each position in the sequence. The position of the three α-helices that form each MYB repeat are marked (Helix 1–3). The asterisks indicate the conserved residues in the MYB domain, whereas the different residues in MYB domain of pea and *Arabidopsis* are marked with triangle marker.

It is well documented that the R2R3-MYB subfamily proteins share a high degree of sequence similarity in the DNA binding domain (DBD) region (Dubos et al., 2010), while sequences outside the DBD region varied greatly in amino acid length and composition. Therefore, to determine the conservation level at each residue position in the R2 and R3 repeats of the R2R3-MYB proteins, sequence logos were generated for pea and *Arabidopsis* (Figure 2). The results showed that the two MYB repeats covered about 105 amino acid residues (including the linker region), with rare deletions or insertions. The R2 and R3 repeats of the MYB domain exhibit different and characteristic amino acid patterns, but are highly conserved between pea and *Arabidopsis*. The R2 repeat possesses three regularly spaced tryptophan (W) residues (Figures 2A,B), which hold together the three helices; however, the R3 repeat contains two highly conserved W residues, which correspond to the second and the third W residues of the R2 repeat respectively, and its first W residue is substituted by phenylalanine (F) or, less frequently, by isoleucine(I), tryptophan(W) or leucine(L) (Figures 2C,D). The residue type-distributions at positions 19, 20, 21, 48 of the R2 repeat and positions 81, 84 of the R3 repeat were apparently different between pea and *Arabidopsis*, and an extra residue was found to be added after the position 21 in the pea R2 repeat (Figures 2A–D). Generally, less conserved residues were found in helix1 and lelix2 of the repeats, while, due to its DNA-recognition function, helix3 of the repeats is more conserved than helix1 and helix2. The above results indicated that the R2R3-MYB DBD domain in pea and *Arabidopsis* was highly conserved, but reflected species differences to a certain extent.

### Analysis of Phylogenetic Relationships, Gene Structure, and Conserved Motifs

**FIGURE 3:**
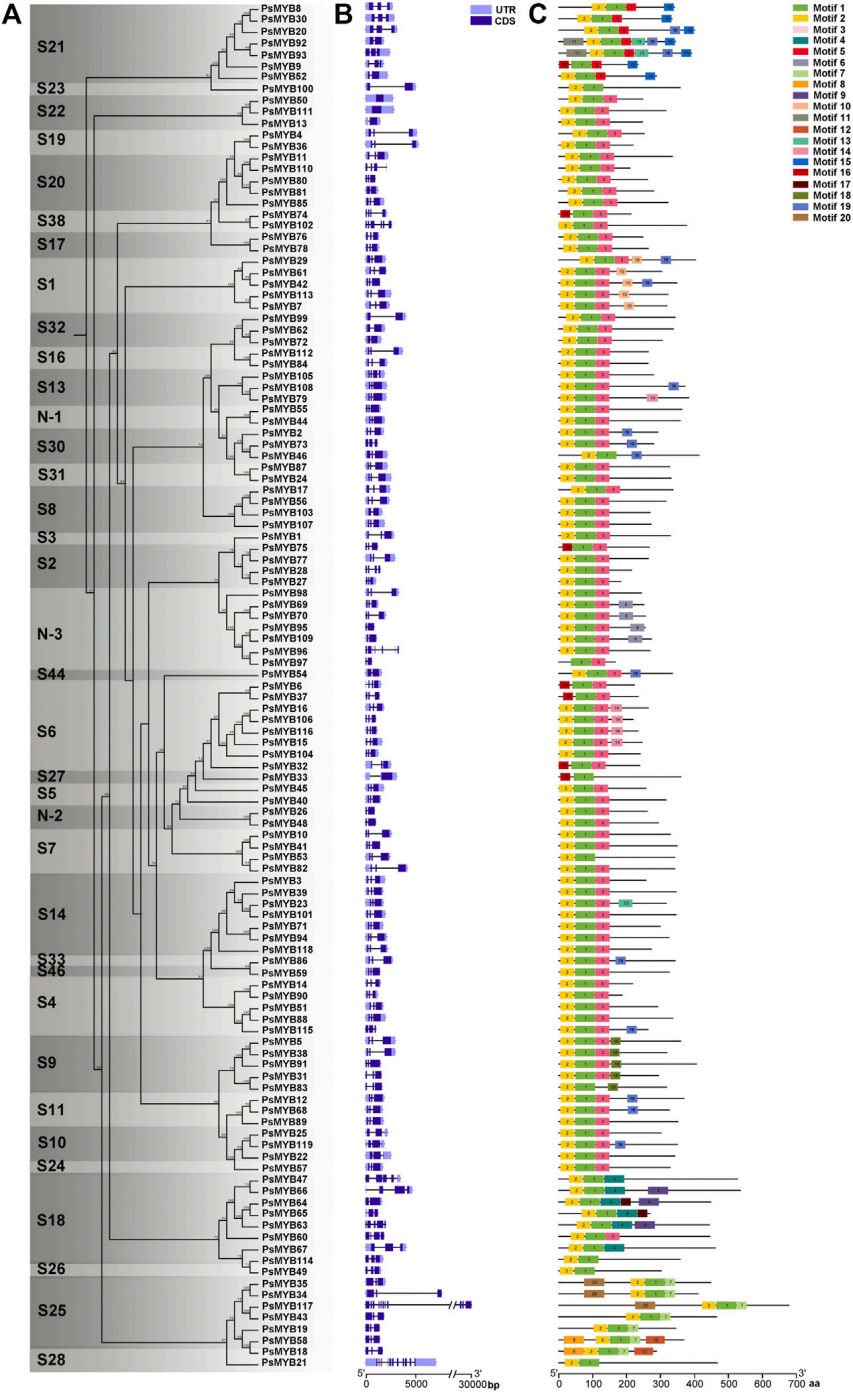
Phylogenetic, gene structure, and Motif analyses of *R2R3-MYB* genes of pea. **(A)** Phylogenetic relationships between R2R3-PsMYBs. The phylogenetic tree (left panel) was constructed with MEGA 6.0 using the Neighbor–Joining (NJ) method with 1,000 bootstrap replicates. **(B)** Gene structure analysis of *R2R3-MYB* genes. Gene structure maps were drawn with the TBtools. The green box, orange box, black lines indicate coding sequence (CDS), the untranslated region (UTR), and the intron, respectively. The scale bar is shown at the bottom. **(C)** Motif analysis of *R2R3-MYB* genes. Twenty conserved Motifs are shown in different colors, the Motif sequence information is provided in Supplementary Figure S1. All Motifs were identified by MEME Version5.1.1 software.

Combined with the phylogenetic tree, intron–exon structure, and conserved Motif analysis of all *R2R3-MYB* genes in pea, we found that the patterns of intron–exon structure and amino acid Motifs were remarkably similar among members within one group but distinct between groups. Most *R2R3-MYB* genomic sequences are less than 1 kb in length with few exceptions just like the longest *PsMYB*117 of 2.5 kb. The exon-intron structure analysis showed that most of these genes comprised 1 or 2 introns, four genes (*PsMYB60*, *74*, *105* and *110*) had 3 introns, two of the subgroup 18 (*PsMYB47* and *63*) had 4 introns, one (*PsMYB102*) had 5 introns, one (*PsMYB96*) had 6 introns, and a maximum of 10 and 11 introns were found in *PsMYB117* and *PsMYB21*, while the subgroup 22 is annotated to be intron less in the coding region (Figures 3A,B). In general, genes within the same subgroups usually had similar gene structure (intron numbers and exon length), suggesting that the exon/intron structures and the phylogenetic relationship between the *R2R3-PsMYB* genes are highly correlated.

To better understand the conservation and diversification of the R2R3-MYB proteins, we analyzed the conserved Motifs between these proteins. A total of 20 conserved amino acid Motifs named Motif 1 to Motif 20 were found, and the number of Motifs for each protein varied from three to six (Figure 3C; Supplementary Figure S1). Similar Motif compositions are shared by R2R3-MYBs which cluster together. All R2R3-PsMYBs contained the Motif 1 which is related to the C-terminal part of the R2 domain (position 39–54) and most of the R3 domain (position 55–98); the Motif 2 was corresponded to most part of the R2 domain (position 1–38) and presented in almost all R2R3-PsMYBs, except that members contained incomplete R2 domain, just like PsMYB18, PsMYB75 and PsMYB97 (Supplementary Figure S2); the N-terminal part of the Motif 3 was corresponded to the C-terminal part of the R3 domain and thus it was also presented in almost all R2R3-PsMYBs. Notably, some members do not have the Motif 3 but alternative Motifs replaced it and members of the same subgroup usually share unique Motif, just like Motif 5 for subgroup 21, Motif 4 for subgroup 18 and Motif 17 for subgroup 25. These results indicated that the most part of the R2 and R3 domains was highly conserved among R2R3-PsMYB members whereas the C-terminal part of the R3 domain is relatively variable but appeared to be subgroup-specific. Many other Motifs serve as characteristics for distinguishing subgroups: Motif 10 for subgroup 1, suggesting that this Motif may be related to abiotic and biotic stress response. Motif 15 for subgroup 21, which mean this Motif may involve in cell wall thickening and lateral organ formation, Motif 18 for subgroup 9, indicating that this Motif may be related in conical epidermal cell outgrowth and trichome branching, most members of subgroup 6 possessed Motifs 14, suggesting that it may related to anthocyanin biosynthesis, most members of the subgroup N-3 possessed Motif 6, which mean that it may play particular role of this new subgroup. These subgroup-specific Motif compositions may be seen as a hint that *R2R3-PsMYB* genes may have been acquired or been lost some functions during the divergence, and the various Motifs may be the reason why different subgroups had different functions.

### Chromosome Locations and Gene Duplication of the *R2R3-MYB* Genes

**FIGURE 4:**
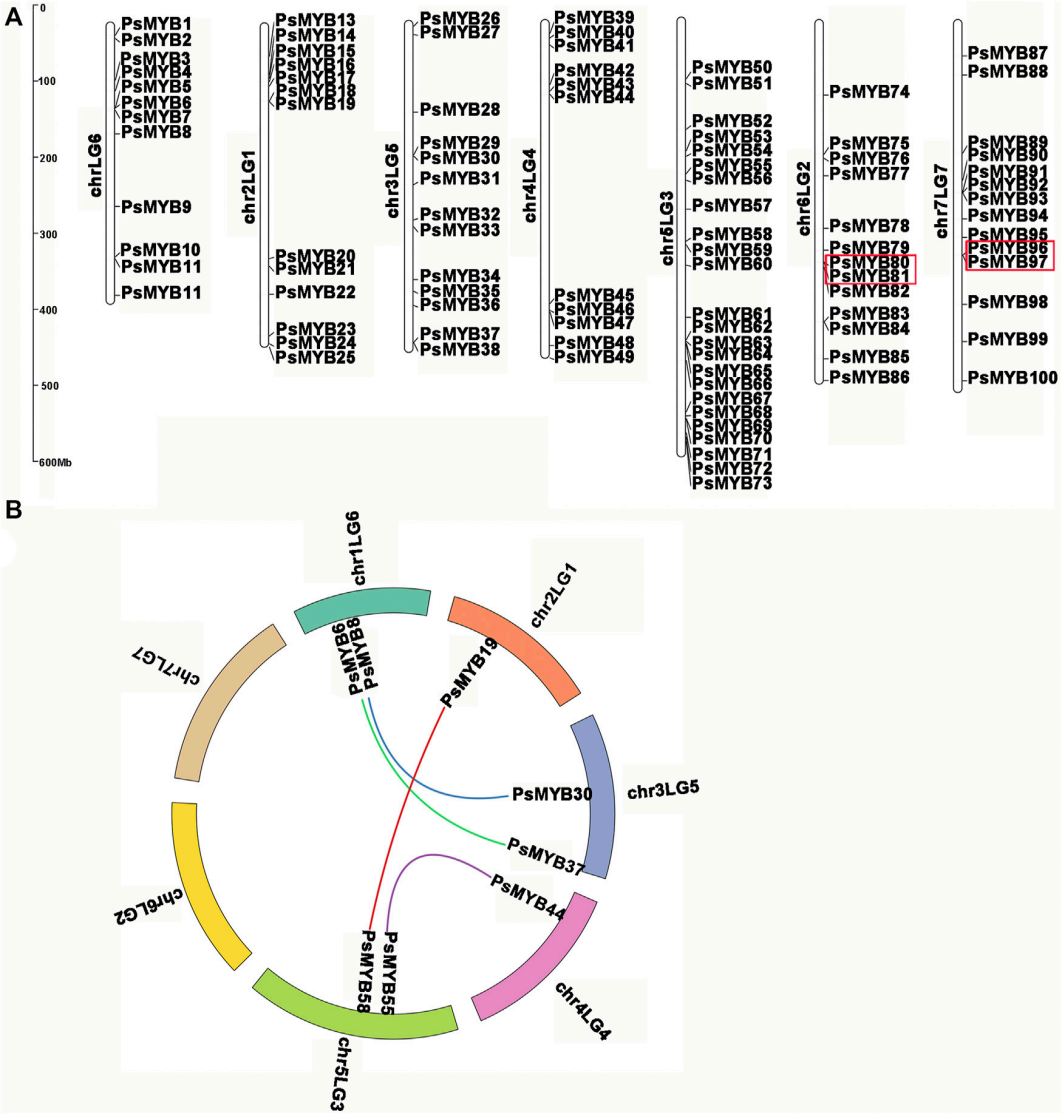
Chromosomal locations and gene duplication of pea *R2R3-MYB*. **(A)** The chromosomal position of each *R2R3-PsMYB* was mapped into the *P*. *sativum* genome. The chromosome number is indicated at the left of each chromosome. The scale is in mega bases (Mb). The tandemly duplicated genes are indicated by red box. **(B)** The segmentally duplicated genes are marked in different color curve lines.

Using the complete pea genome sequence, we plotted the chromosomal locations of all *R2R3-PsMYB* genes. The chromosome numbering was assigned according to the previous work (Kreplak et al., 2019). A total of 100 *R2R3-PsMYB* genes were unevenly distributed throughout the 7 chromosomes (Figure 4A): the chromosome 5 had the largest number of *R2R3-PsMYB* genes (*n* = 25), while the chromosome 1 had the smallest number (*n* = 13). The other 19 *R2R3-PsMYB* genes were found to be located on 19 unanchored scaffolds (Supplementary Table S3). To understand the mechanism of expansion of the *R2R3-MYB* genes in pea genome, we investigated tandem and segmental duplication events by genome synteny analysis. The result indicated that two gene pairs were produced by tandem duplication events, one pair on chromosome 6 (*PsMYB80* and *PsMYB81*) and another on chromosome 7 (*PsMYB96* and *PsMYB97*) (Supplementary Table S4), while four putative paralogous gene pairs locating on chromosomes 1, 2, 3, 4 and 5 were resulted from segmental duplications, and they are belonged to four different subgroups (S6, S21, S25 and N-1) (Figure 4B; Supplementary Table S4). However, no whole-genome duplication has been documented for the *R2R3-MYB* genes family expansion in pea genome.

The Ka/Ks ratios (where Ka = nonsynonymous substitutions per site, and Ks = synonymous substitutions per site) of these duplicated pairs were estimated and were tested statistically. Generally, Ka/Ks > 1 indicates positive selection, Ka/Ks = 1 indicates neutral selection, while Ka/Ks <1 indicates negative or purifying selection. In this study, Ka/Ks ratios for six *R2R3-PsMYB* genes pairs were no larger than 0.5, ranging from 0.17 to 0.50 with an average of 0.35 (Supplementary Table S4). The results indicated that these segmental and tandem duplicates have been subject to strong functional constraints during the process of evolution and they may have mainly experienced strong purifying selection pressure with limited functional divergence after duplication events.

### Morphological Anatomy of Pea Flowers During Development

**FIGURE 5:**
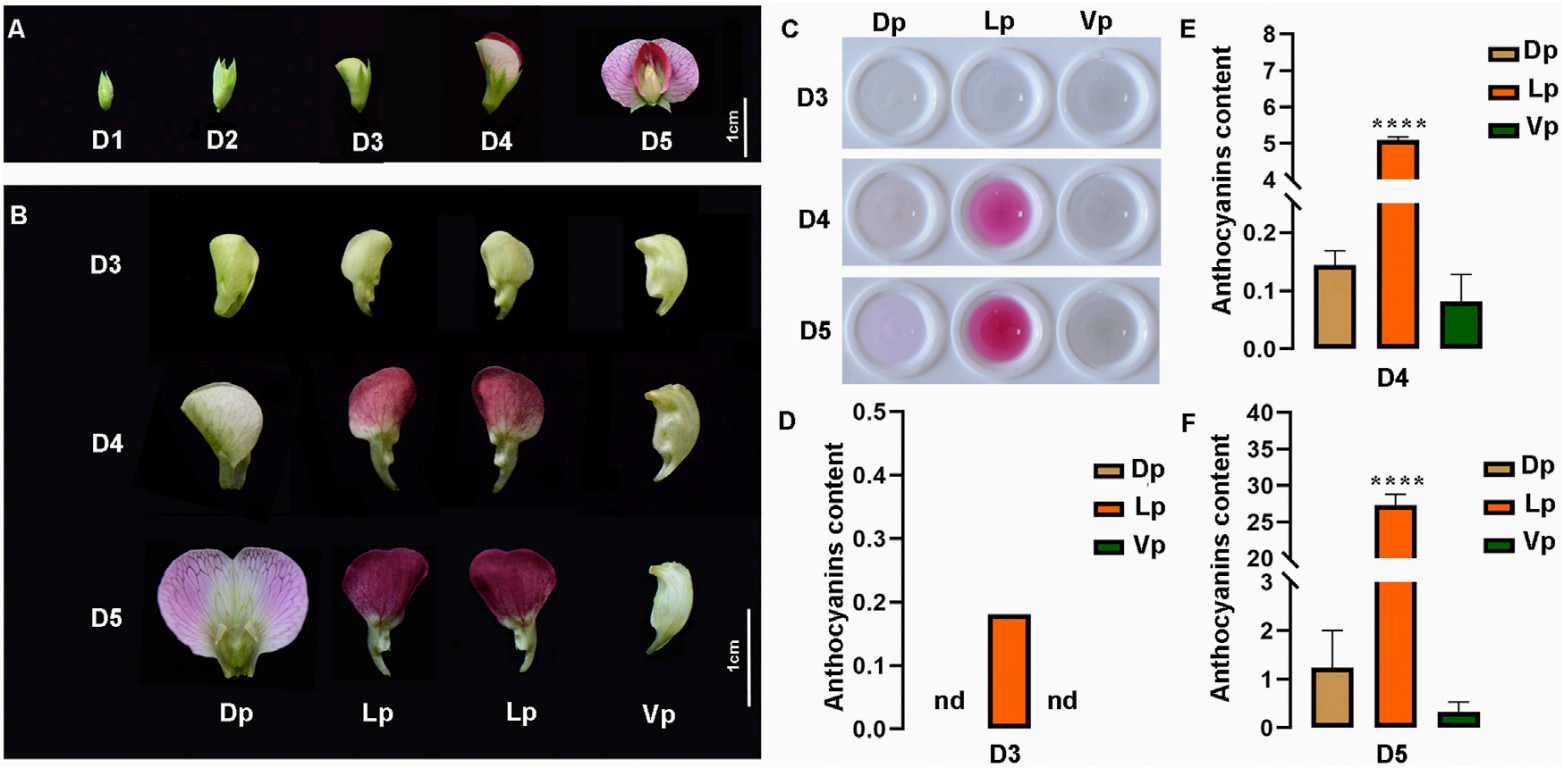
Characteristics of pea flowers during different developmental stages. **(A)** Morphological analysis of pea flowers. Bub length < 0.6 cm, 0.6–0.7 cm, 0.9–1 cm, 1.3–1.9 cm, > 2 cm are defined as developmental stage D1 to D5, respectively. **(B)** Morphological anatomy of pea flowers during D3 to D5. Dp, Dorsal petals; Lp, Lateral petals; Vp, Ventral petals. nd, not detected. **(C)** The crude pigment extraction from Dp, Lp, and Vp at D3 to D5. **(D–F)** The total anthocyanin contents in the Dp, Lp, and Vp of D3 **(D)**, D4 **(E)**, D5 **(F)** were detected by ultraviolet spectrophotometer, computed as (A_530_-0.25 × A_657_)/FW (g^−1^ FW). The data are the mean ± SD from three biological replicates. **p < 0.05. ****p < 0.0001, Ordinary one-way ANOVA; nd, not detected; Bar = 1 cm.

Here, according to the length of flower buds, five developmental stages, which cover the phase of the anthocyanin pigment accumulation, were defined as follows: D1 (<0.6 cm), D2 (0.6–0.7 cm), D3 (0.9–1 cm), D4 (1.3–1.9 cm), and D5 (> 2 cm). The pea flower is asymmetric along its dorsoventral axis, having two fused dorsal petals (Dp), two lateral petals (Lp), and one ventral petal (Vp). From D1 to D3, no red pigment production was observed in any tissues of the flowers (Figure 5A). From D3 to D5, the color of the Dp changed progressively from green to light-purple, and the Lp from green to deep-red, but no obvious pigment formation was observed in the Vp (Figures 5A,B). Based on the color analysis of the pea flowers, we then extracted the petal pigments and confirmed that the pigments increased gradually with the flower maturation and mainly accumulated in the Lp, followed by the Dp, but no pigment observed in Vp during any developmental stages (Figure 5C). Then, quantitative analysis of the anthocyanin content of the extracts clearly showed the spatio-temporal pattern of the pigment accumulation during the flower development. The anthocyanins of the Lp tissues can be detected as early as the D3 stage, but no anthocyanin detected in the Dp and Vp in this stage. At stage D4, the Lp contained a high accumulation of anthocyanins, while a slight content of pigments was also detected in the Dp and Vp (Figures 5D–F). At stage D5 when the flower was fully opened, the anthocyanin content of each petal part reached its highest level. These results suggested that anthocyanins increased during flower development and the pigment distribution among the petals was unbalanced.

### Identification of R2R3-MYBs Involved in the Regulation of Anthocyanin Biosynthesis in Pea

**FIGURE 6:**
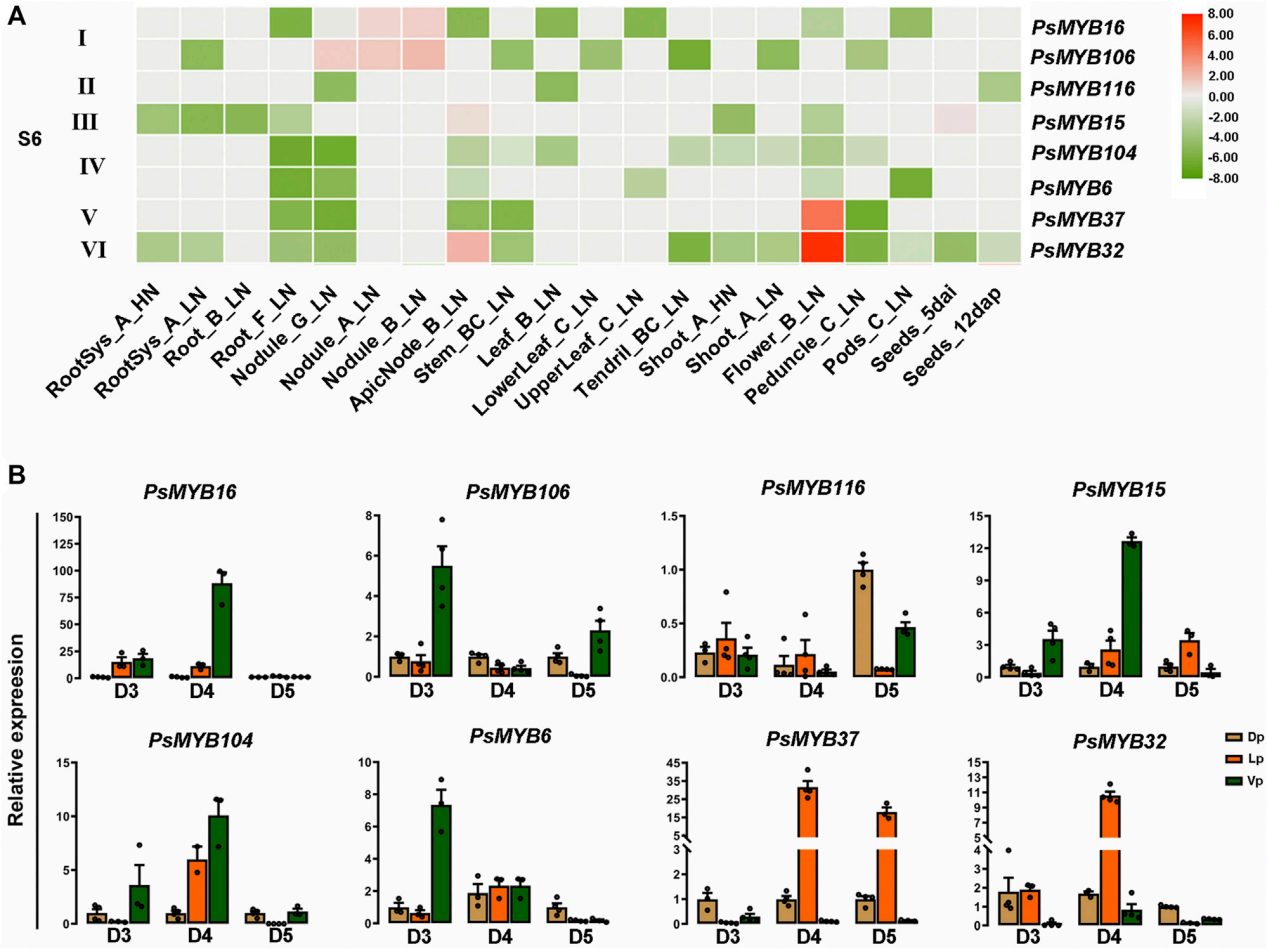
Identification of R2R3-MYB candidates involved in pigment biosynthesis. **(A)** Heat-map showing the expression level of genes from S6 subgroup under different nutritive conditions and different stages based on RNA-seq data. Normalized log2 transformed values were used with hierarchical clustering represented by the color scale (−9–8). Green indicates low expression, and red indicates high expression. HN, High N (14 mM N). LN, Low N (0.625 mM N). **(B)** The expression profiles of S6 R2R3-MYB family during flower development were confirmed using qRT-PCR. The data are the mean ± SD from three biological replicates. Dp, Dorsal petals; Lp, Lateral petals; Vp, Ventral petals.

The phylogenetic tree suggested that eight R2R3-MYBs from pea belonged to subgroup 6 of the R2R3-MYBs, which is related to PAP1, a positive regulator of the anthocyanin biosynthesis. We then examine the gene expression patterns of the subgroup 6 genes to infer the putative functions. Based on the public expression data of the *R2R3-MYBs* from subgroup 6 (Supplementary Table S5), relative expression heat-map of the subgroup 6 members was generated. The results showed that the *R2R3-PsMYBs* of the subgroup 6 could be classified into six types based on their expression patterns: type I *R2R3-MYBs* are specially expressed in the nodule, including *PsMYB16* and *PsMYB106*; the type II gene (*PsMYB116*) and the type IV *R2R3-MYB* genes, including *PsMYB104* and *PsMYB6*, are lowly expressed in all tissues but displayed different patterns; the type III *R2R3-MYB* (*PsMYB15*) has a moderate expression level in node and seeds while a low expression level in other tissues; both the type V (*PsMYB37*) and VI (*PsMYB32*) *R2R3-MYBs* have a highest expression level in flowers and the type V gene also has a relatively high expression level in node (Figure 6A). The result suggested that *PsMYB37* and *PsMYB32* were highly expressed in flowers while other subgroup 6 members showed a relatively low or weak expression level. We then checked the expression profiles of these genes in different petals of flowers at different developmental stages by quantitative real-time PCR (qRT–PCR) to determine the associations between gene expression and anthocyanin level (Figure 6B). Five members (*PsMYB16*, *106*, *15*, *104* and 6) were found to be highly expressed in the Vp tissue, but their predominant expression was observed at particular development stage. In detail, three members (*PsMYB16*, *15* and *104*) had the predominant expression at D4 stage, the other two members (*PsMYB106* and 6) had the predominant expression at D3 stage, but noticeably, the *PsMYB106* also showed a relatively high expression level in the Vp tissue of the flower at D5 stage. Only one member, namely *PsMYB116*, had the highest expression level in the Dp tissue and a relatively high expression level in the Vp tissue of the flower at D5 stage. The other two members *PsMYB37* and *PsMYB32*, both are specifically or highly expressed in the Lp tissues, but the *PsMYB37* had the predominant expression at D4 and D5 stage, while the *PsMYB32* had the predominant expression at D5 stage. In conclusion, the high expression of *PsMYB16*, *106*, *15*, *104* and *6* in the Vp tissue was accompanied by the low anthocyanin accumulation, and the high expression level of *PsMYB37* and *PsMYB32* in the Lp tissue were consistent with the deepening of anthocyanin accumulation and transcriptome data, which suggesting that they may be involved in regulating of anthocyanin biosynthesis, and can be screened as negative and positive candidate genes for anthocyanin accumulation in pea flower petals. The above results suggested that *R2R3-MYB* genes are involved in specific functions with organ- or tissue-specific expression.

## Discussion

Anthocyanins are beneficial to human health, presumably as natural antioxidant for preventing of cardiovascular and cerebrovascular diseases (Zafra-Stone et al., 2007; Mattioli et al., 2020). Recently, engineering metabolic pathways for biofortification of crops to produce “healthier foods” has attracted increasing research attention, and some achievements have been made, such as anthocyanin-enriched “Purple Tomatoes” and “Purple Endosperm Rice” (Butelli et al., 2008; Zhu et al., 2017). Pea accumulates anthocyanins mainly in flowers but not in seed which is edible, thus it could be a good reason for enhancing anthocyanin content in pea seeds. R2R3-MYBs are considered to positively regulate anthocyanins-related genes by interacting with bHLH and WD40 to form the MBW complexes, and thus affect the spatiotemporal accumulation of anthocyanins (Liu et al., 2015; Zhu et al., 2020; Yan et al., 2021).

In this study, we identified 119 R2R3-MYB transcription factors in pea (Supplementary Table S1), and we did not observe large-scale duplication events in pea genome, furthermore, some R2R3-MYBs were evolutionarily highly conserved (Figure 4; Supplementary Table S4). It seems that the numbers of the R2R3-MYB family were not strictly correlate to the species genome size considering the following examples: there were 119 genes in pea with a large genome size (3.9 Gb) (Kreplak et al., 2019), 25 genes in barley with a 5.1 Gb genome size (Mayer et al., 2012; Tombuloglu et al., 2013), 150 genes in *Medicago truncatula* (500 Mb) (Young et al., 2011; Li et al., 2019), and 126 genes in *Arabidopsis thaliana* (125 Mb) (Kaul et al., 2000; Ralf Stracke, 2001). These results were consistent with previous findings that the expansion of R2R3-MYBs during evolution was not dependent on whole-genome duplication events but on multiple gene duplications, which exhibited asymmetry between subfamilies and clades and caused functional diversification to handle the diversity and complexity of plants (Jiang and Rao, 2020; Wu et al., 2022).

Anthocyanin biosynthesis is mainly determined by the expression levels of structure genes, and R2R3-MYBs play the significant role in controlling the expressions of these genes thus directly influence the color pattern of flowers (Li, 2014; Zhao and Tao, 2015). The inheritance of flower color in pea has been studied for more than a century (Statham et al., 1972a; Hellens et al., 2010; Moreau et al., 2012), but the spatio-temporal pattern of the pigment accumulation and the genes corresponding to this biological process has been poorly investigated. In our study, PsMYB116, PsMYB37, and PsMYB32 were clustered with PAP1/MYB75 and PAP2/MYB90, which have been shown to promote anthocyanin accumulation in *Arabidopsis* (Teng et al., 2005; Gonzalez et al., 2008). According to the analysis of morphological anatomy and expression patterns, our research divided the process of the color pattern formation of pea flowers into five stages. From D1 to D2, the anthocyanin-related genes were inactivated, resulting in no pigment production in flowers (Figures 5, 6). At D3 stage, flowers are still green in color, but a very weak pigment production was detected in the Lp tissues, indicating the anthocyanins biosynthesis pathway might begin to be activated. At D4 stage, the Lp tissues showed a high level of pigment production with the color change from green to purple, but trace pigment production was also detected in the Dp and Vp tissues despite no color change observed, indicating the anthocyanins biosynthesis pathway was activated in all petals but in a complex spatio-temporal manner to color the petals. In this stage, *PsMYB37* and *PsMYB32* may be of a positive correlation with the anthocyanin accumulation in the Lp tissues. At the final stage D5, the anthocyanins biosynthesis pathway would be in a continuously activated state in the Lp and Dp tissues, resulting in the deep purple and light purple colors, respectively. In this stage, *PsMYB116* may be the key regulator to cause the purple coloration in the Dp tissues, and notably, *PsMYB37* might play a leading role in maintaining purple color in the Lp tissues at D5 stage rather than *PsMYB32*. R2R3-MYBs usually combine with the bHLH protein to control the activities of sets of downstream genes, such as *F3*′*H*, *F3*′*5*′*H*, *DFR*, *ANS*, *LAR*, *3GGT*, *UGT78G1*, *MATE*, and *GST*, thus determining the spatiotemporal accumulation of anthocyanins (Jun et al., 2015; Li et al., 2016; Zhou et al., 2018; Lu et al., 2021; Wang et al., 2021). According to the previous study (Hellens et al., 2010), we confirmed that the *A* gene encoding a PsbHLH transcription factor plays a key role in the regulation of anthocyanin accumulation in petals, especially in the Lp but not in the Dp and Vp tissues (Supplementary Figure S3; Supplementary Table S6). It is worth noting that, PsMYB37 and PsMYB32 had the same Motifs (Motifs 1, 3, 16) in the amino acids sequence, which is different from the regions existed in the PsMYB116(Motifs 1, 2, 3, 14) (Figure 3), indicating that they might be activate by different activators, thus, may have caused the different spatiotemporal expressions between PsMYB37, PsMYB32, and PsMYB116. Based on our findings, we speculated that, PsMYB116, PsMYB37, and PsMYB32 might couple with the PsbHLH to collectively regulate spatiotemporal expression levels of anthocyanin-associated genes, which resulted in the unbalanced pigment distribution pattern of pea flowers, but their true functions remain to be further explored in the future. These findings can enhance the understanding the mechanism of flower color pattern formation, and provide a new insight towards to the metabolic engineering of anthocyanins biosynthesis pathways for biofortification of pea to produce “healthier foods” for humans.

## Materials and Methods

### Plant Materials

JI2822 accession was used for all the experiments and was grown in a climate-controlled greenhouse with a 16 h light and 8 h dark cycle at 23°C, and 50%–60% relative humidity.

### RNA Isolation, Quantitative Real-Time, and Data Analysis

To show the tissue-specific expression profiles of S6 genes under different nutritive stresses, we counted the value of the expression based on RNA-seq data reported previously (Alves-Carvalho et al., 2015). Normalized log2 transformed values were used to draw the heatmap via TBtools (Chen and Xia, 2020). Furthermore, to determine the accumulation of anthocyanin biosynthesis in flower, fresh flower buds were prepared from five development stages D1 (<0.6 cm), D2 (0.6–0.7 cm), D3 (0.9–1 cm), D4 (1.3–1.9 cm), D5 (> 2 cm). Then, the samples were immediately frozen in liquid nitrogen for RNA isolation. Total RNA was prepared using RnaEx™ Total RNA isolation (Shanghai GENEray Biotech Co., Ltd.), and cDNA was reverse-transcribed from 5 μg of total RNA with the HiScript®II 1st Strand cDNA Synthesis Kit (+gDNA wiper) (Nanjing Vazyme Biotech Co., Ltd.). In qRT–PCR experiments, each result was obtained from three experiments using independent RNA samples. The reaction parameters were as follows: 95°C for 1°min, followed by 40 cycles of 95°C for 10 s, 55°C for 5°s and 72°C for 15 s. Then, a melting curve was generated from 65°C to 95°C. One-way ANOVA was performed using GraphPad Prism 8 software. The primers used for qRT–PCR analysis were designed by Primer designing tool in NCBI (https:// www.ncbi.nlm.nih.gov/tools/primer-blast/) and listed in Supplementary Table S7. The pea *ACTIN* gene *PsACT* was used as the internal control for each PCR experiment (Sussmilch et al., 2015). The expression level of each *R2R3-MYB* gene was calculated using the 2^−∆∆CT^ method (Livak and Schmittgen, 2001). All analyses were repeated three times using biological replicates.

### Sequence Retrieval and Phylogenetic Analysis

The pea and *Arabidopsis* genome sequences were obtained from the Pea Genome project website (Kreplak et al., 2019) (https://urgi.versailles.inra.fr/Species/Pisum/Pea-Genome-project) and TAIR 10 (http://www.Arabidopsis.org/), respectively. To identify the maximum number of MYB domain-containing sequences in pea, a genome-wide Hidden Markov Model (HMM) search was first performed by local version of the HMMER v3.2.1 program with the default value (Ghahramani, 2001). The HMM model (PF00249) of the MYB DNA-binding domain was downloaded from Pfam (http://pfam.sanger.ac.uk/) (El-Gebali et al., 2019). There were 333 proteins considered putative MYB genes. Second, to confirm the obtained amino acids, the 333 candidate *MYB* genes were manually analyzed in the Pfam database, and 126 MYB proteins were inspected to ensure that the putative gene models contained two MYB repeats. Third, multiple sequence alignment of all identified R2R3-MYB proteins was performed using ClustalX with default parameters to identify conserved MYB repeat sequences, and 119 R2R3-MYBs were obtained. The sequences with incomplete R2R3-MYB domain structures were discarded. Furthermore, the basic physicochemical parameters of R2R3-MYB proteins, such as the protein length, molecular weight (Mw), and isoelectric point (pI), were predicted by the ExPaSy Proteomics Server (Gasteiger et al., 2003).

A phylogenetic tree of the full-length amino acid sequences of R2R3-MYBs was constructed using the neighbor-joining algorithm with default parameters and 1,000 bootstrap replicates in MEGA 5.0 software (Tamura et al., 2011). The phylogenetic tree was subsequently visualized using iTOL (https://itol.embl.de/) (Letunic and Bork, 2019).

### Conservation Analysis of R2R3-MYB Proteins

Sequences of R2 and R3 repeats of 119 pea R2R3-MYB proteins were first aligned with ClustalX (Larkin et al., 2007), and the sequence logos of R2 and R3 repeats were generated by submitting the multiple sequences to http://weblogo (Crooks et al., 2004).

### Gene Structure, Chromosomal Location, and Gene Duplication Analysis of *R2R3-MYB* Genes

The DNA and cDNA sequences corresponding to each predicted *R2R3-MYB* gene were downloaded from the pea genome database. The intron/exon structures and chromosome locations of *R2R3-MYB* genes were drawn by TBtools software (Chen et al., 2020). The conserved Motifs were predicted by MEME Version 5.1.1 (http://meme-suite.org/tools/meme) (Bailey et al., 2009). The following parameters were used: any number of repetitions, the maximum number of Motifs-20, and optimum width from 30 to 60. For gene duplication event, synteny relatonships, Ka, Ks, and their ratio in all of the duplicated *R2R3-MYB* gene pairs were estimated using TBtools with default parameters (Chen et al., 2020). It is worth noting that only genes located on chromosomes were used to analyze duplications and synteny relationships.

### Measurement of the Total Anthocyanins in Pea Flower

Fresh flower tissues were collected and ground into power in liquid nitrogen, followed by extraction with 1 ml anthocyanin extraction (0.1% hydrochloric acid/methanol). These samples were vortexed for 1 min and then extracted at 4°C for 1 h. The solution was centrifuged at 12,000 g for 5 min to collect the supernatant, and the residue was extracted with extraction solution 1, 2 times until the supernatant turned colorless. The total anthocyanin contents were computed as (A530-0.25 × A657)/FW(g^−1^ FW).

## Data Availability

The original contributions presented in the study are included in the article/Supplementary Material, further inquiries can be directed to the corresponding authors.

## Supplementary Material

Supplementary Figure S1 Conserved Motifs and predicted structures of R2R3-MYB genes in pea.

Supplementary Figure S2 Multiple alignment of R2R3-MYB domains of 119 pea R2R3-MYB proteins. ClustalW amino acid sequence alignment. The black arrowheads at the top indicate the conserved tryptophan (W) or phenylalanine (F) residues. Red letters represent proteins with incomplete R2 domains. Position numbers at the top correspond to those given in Figure 2.

Supplementary Figure S3 The expression pattern of PsbHLH. **(A)** Heat-map showing the expression level of PsbHLH under different nutritive conditions and different stages based on RNA-seq data. Normalized log2 transformed values were used with hierarchical clustering represented by the color scale (−9–8). Green indicates low expression, and red indicates high expression. HN, High N (14 mM N). LN, Low N (0.625 mM N). **(B)** The expression profiles of PsbHLH during flower development were confirmed using qRT-PCR. The data are the mean ±SD from three biological replicates. Dp, Dorsal petals; Lp, Lateral petals; Vp, Ventral petals.
