# Supplementary figures and images for: The Pea R2R3-MYB Gene Family and Its Role in Anthocyanin Biosynthesis in Flowers
Source: Front Genet. 2022 Jul 6;13:936051. doi: 10.3389/fgene.2022.936051 (PMC9299958; doi:10.3389/fgene.2022.936051)

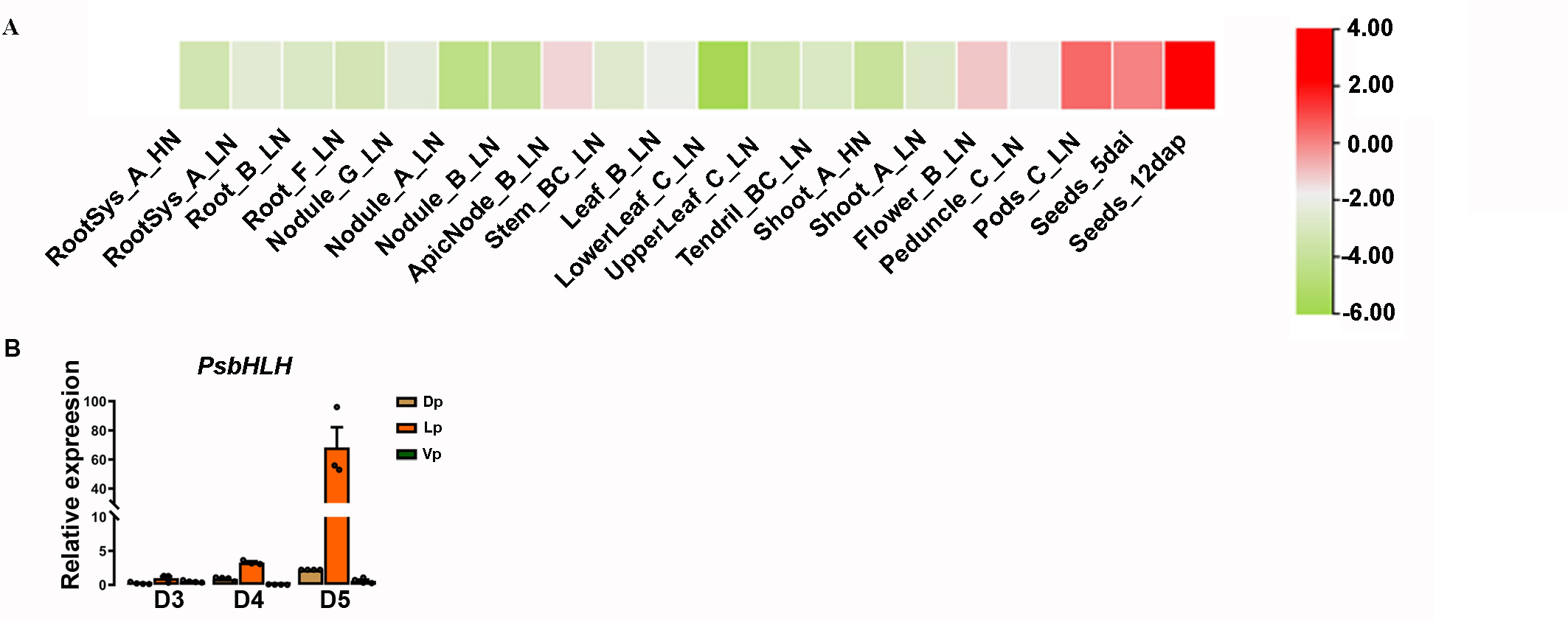

Supplement: Supplementary file 1 [file Image3.jpg]

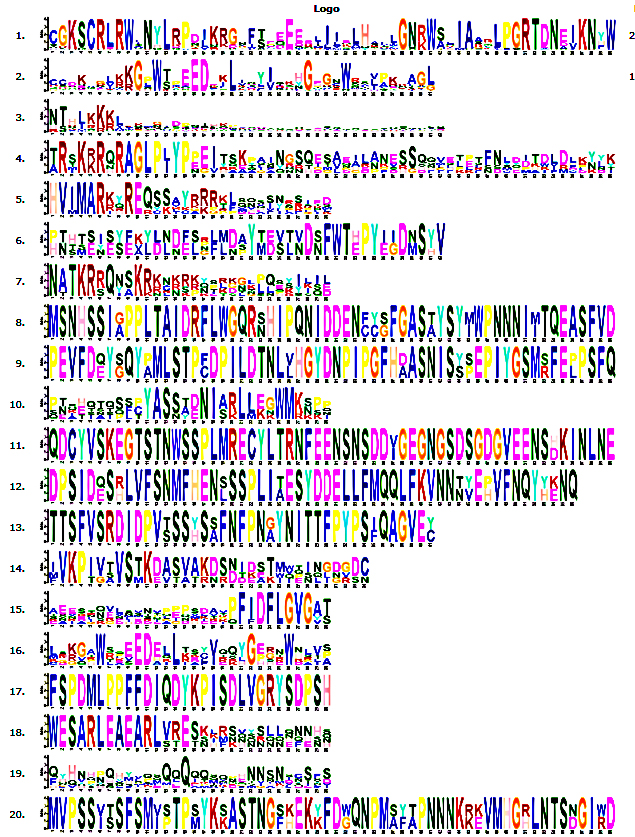

Supplement: Supplementary file 2 [file Image1.JPEG]

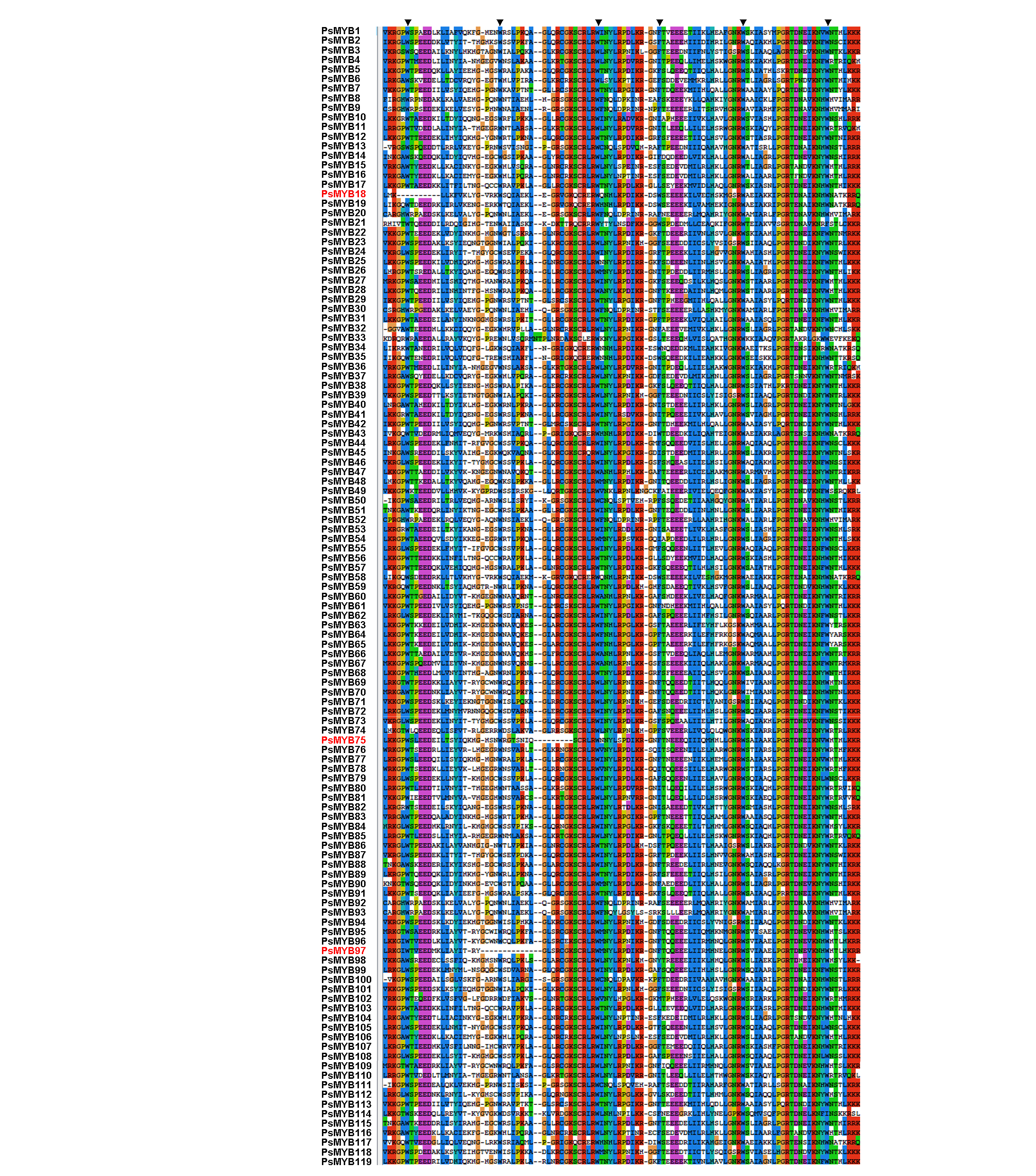

Supplement: Supplementary file 3 [file Image2.JPEG]
